# Supplementary material for: Out-of-Plane Magnetic Anisotropy in Ordered Ensembles of FeyN Nanocrystals Embedded in GaN
Source: Materials (Basel). 2020 Jul 24;13(15):3294. doi: 10.3390/ma13153294 (PMC7436053; doi:10.3390/ma13153294)
Supplement: Supplementary file 1 [file materials-13-03294-s001.pdf]

materials

Supplementary Materials: Out-of-plane Magnetic Anisotropy in Ordered Ensembles of Fe_yN Nanocrystals Embedded in GaN

Andrea Navarro-Quezada [1,*], Katarzyna Gas [2], Tia Truglas [3], Viola Bauernfeind [3], Margherita Matzer [1], Dominik Kreil [4], Andreas Ney [1], Heiko Groiss [3], Maciej Sawicki [2] and Alberta Bonanni [1]

1 Institute of Semiconductor and Solid-State Physics, Johannes Kepler University Linz, Altenberger Str. 69, 4040 Linz, Austria; margherita.matzer@jku.at (M.M.); andreas.ney@jku.at (A.N.); alberta.bonanni@jku.at (A.B.)
2 Institute of Physics, Polish Academy of Sciences, Aleja Lotnikow 32/46, 02668 Warsaw, Poland; kgas@ifpan.edu.pl (K.G.); mikes@ifpan.edu.pl (M.S.)
3 Christian Doppler Laboratory for Nanoscale Phase Transformations, Johannes Kepler University Linz, Altenberger Str. 69, 4040 Linz, Austria; Tia.truglas@jku.at (T.T.); viola.bauernfeind@speed.at (V.B.); heiko.groiss@jku.at (H.G.)
4 Institute of Theoretical Physics, Johannes Kepler University Linz, Altenberger Str. 69, 4040 Linz, Austria; dominik.kreil@jku.at
* Correspondence: andrea.navarro-quezada@jku.at; Tel.: +43-732-2468-9622

The chemical composition of the embedded NCs and their surrounding has been analysed *via* energy dispersive x-ray spectroscopy (EDX) line-scans measured in scanning TEM (STEM) imaging mode. The comparison between the line-scans acquired from areas with and without NCs for the reference layer and for the layer grown on the $Al_{0.41}$ $Ga_{0.59}N$ buffer are shown in Figure S1. The relative concentration of Ga, N, Al and Fe *vs.* depth from the sample surface is reported. An Al background of about 1–3% is detected in all scans that originates from scattered electrons that excite x-rays in the Al_2O_3 substrate or from redeposition of material from the substrate onto the GaδFeN layer during ion milling, a necessary step in the TEM sample preparation. Therefore, diffusion of Al into the GaδFeN layer for the sample grown on the $Al_{0.41}Ga_{0.59}N$ buffer can be ruled out. The relative Fe concentration is below the detection limit of the EDX throughout the buffer layer for both displayed samples.

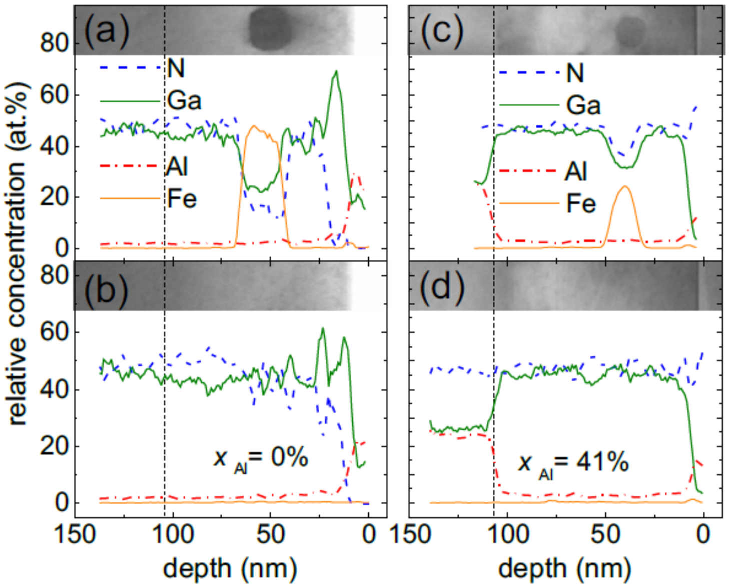

Figure S1. Relative atomic concentration of Ga (thick —), N (- -), Al (-.-) and Fe (—) obtained from EDX. line-scans acquired from areas with (top) and without (bottom) an embedded NC, respectively,

for the reference sample (**a**) and (**b**), and the GaδFeN/$Al_{0.41}Ga_{0.59}N$ sample (**c**) and (**d**). The vertical dashed lines mark the interface between buffer and GaδFeN layer.

Angular-dependent ferromagnetic resonance (FMR) measurements acquired at room temperature are presented in Figure S2. The FMR resonance lines for the investigated layers emerge at a resonance field of 290–315 mT in agreement with the position of the FMR line previously obtained for γ'-$Ga_yFe_{4-y}N$ NCs embedded in GaN [1]. The magnetic field lies in the film plane for $\theta = 0°$ and perpendicular to the plane at $\theta = 90°$.

The angular-dependent FMR data corresponding to the NCs in the GaδFeN layer grown on the $Al_{0.1}Ga_{0.9}N$ buffer, reported in Figure S2, present an uniaxial $\sin^2(\theta)$ dependence of the resonance field in the out-of plane configuration, similar to the one previously observed for γ'-$Ga_yFe_{4-y}N$ NCs in GaδFeN layers grown on GaN [1,2]. In contrast to previous studies, there is a combination of a four-fold, a six-fold and a two-fold components in the in-plane ($\theta = 0°$) angular dependence. This is consistent with the presence of γ'-$Ga_yFe_{4-y}N$ and ε-Fe_3N NCs in the layer. Fitting of the four-fold component yields a saturation magnetization value of 1500 ± 200 emu/cm^3, which is in good agreement with the values obtained for the reference sample from SQUID magnetometry and with values reported for γ'-Fe_4N powders [3] and thin films [4]. The fit also yields an anisotropy constant of $K_1 = (2.8 \pm 0.2) \times 10^4$ J/m^3 for the cubic NCs.

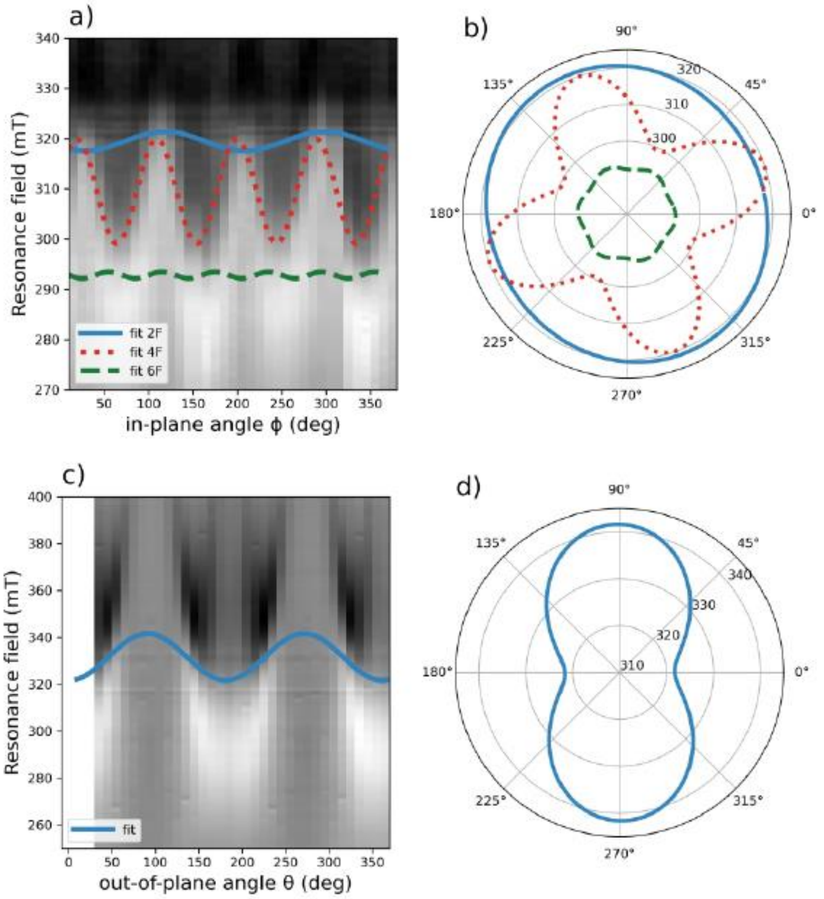

Figure 2. Angular dependence maps and polar plots of the FMR for the GaδFeN/$Al_{0.22}Ga_{0.78}N$ sample with fitted symmetry components (lines) for: (**a**) and (**b**) in-plane angle φ, and (**c**) and (**d**) out-of-plane angle θ.

References

1. Grois, A.; Devillers, T.; 27 Li, T.; Bonanni, A. Planar array of self-assembled $Ga_xFe_{4-x}N$ nanocrystals in GaN: magnetic anisotropy determined via ferromagnetic resonance. *Nanotechnology* **2014**, *25*, 395704.

2. Navarro-Quezada, A.; Aiglinger, M.; Faina, B.; Gas, K.; Matzer, M.; Li, T.; Adhikari, R.; Sawicki, M.; Bonanni, A. Magnetotransport in phase-separated (Ga,Fe)N with γ'- $Ga_yFe_{4-y}N$ nanocrystals. *Phys. Rev. B* **2019**, *99*, 085201.
3. Coey, J.; Smith, P. Magnetic nitrides. *J. Magn. Magn. Mater.* **1999**, *200*, 405–424.
4. Chen, S.K.; Jin, S.; Tiefel, T.H.; Hsieh, Y.F.; Gyorgy, E.M.; Johnson, D.W. Magnetic properties and microstructure of Fe_4N and $(Fe,Ni)_4N$. *J. Appl. Phys*. **1991**, *70*, 6247–6249.

© 2020 by the authors. Submitted for possible open access publication under the terms and conditions of the Creative Commons Attribution (CC BY) license (http://creativecommons.org/licenses/by/4.0/).
